# Nanofabrication of chitosan-based dressing to treat the infected wounds: *in vitro* and *in vivo* evaluations

**DOI:** 10.2144/fsoa-2023-0077

**Published:** 2024-05-15

**Authors:** Maryam Yousefi, Ramin Ghahremanzadeh, Mohammad-Reza Nejadmoghaddam, Fatemeh Yazdi Samadi, Somayeh Najafzadeh, Fereshteh Mohammadi Fatideh, Zohreh Mohammadi, Arash Minai-Tehrani

**Affiliations:** 1 Nanobiotechnology Research Center, Avicenna Research Institute, ACECR, Tehran, 1983969412, Iran; 2 Department of Pharmaceutics & Pharmaceutical Nanotechnology, School of Pharmacy, Iran University of Medical Sciences, Tehran, 1475886973, Iran

**Keywords:** chitosan, film dressing, graphene oxide, infected wounds, zinc oxide

## Abstract

**Aim:** Here, an innovative kind of antibacterial nanocomposite film is developed by incorporating graphene oxide and zinc oxide into chitosan matrix. **Materials & methods:** Our dressing was fabricated using the solution casting method. Fourier transform infrared spectra and TGA-DTG clearly confirmed the structure of film dressing. **Results & conclusion:** Our results showed the tensile strength and elongation at the break of the films were 20.1 ± 0.7 MPa and 36 ± 10%, respectively. Our fabricated film could absorb at least three-times the fluid of its dry weight while being biocompatible, antibacterial, non-irritant and non-allergic. In addition, it accelerated the healing process of infected wounds by regulating epithelium thickness and the number of inflammatory cells, thus it may be useful for direct application to damaged infected wounds.

## Plain language summary

In this study, an innovative kind of antibacterial nanocomposite film is developed by incorporating graphene oxide and zinc oxide into chitosan matrix. Our antibacterial wound dressing was fabricated using the solution casting method. Our fabricated film could absorb at least three-times the fluid of its dry weight while being biocompatible, antibacterial, non-irritant and non-allergic. In addition, our film accelerated the healing process of infected wounds by regulating epithelium thickness and the number of inflammatory cells. thus it may be useful for direct application to damaged infected wounds.

## Abstract (plain language summary)

Production of an innovative kind of antibacterial nanocomposite film by incorporating graphene oxide and zinc oxide into chitosan matrix.

Our fabricated film could absorb at least three-times the fluid of its dry weight while being biocompatible, antibacterial, non-irritant and non-allergic.

Our nanocomposite film displayed a great potential as a novel multifunctional wound dressing application.

## Graphical abstract

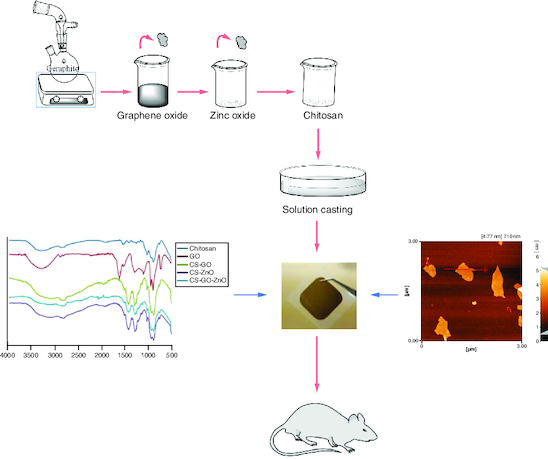

Generally, the number of individuals with chronic or complicated wounds is growing and thus the burden of treating them is rising quickly and is considered to be costly, in terms of both time and resources that may be needed. One of the main medical options for the treatment of wounds is the use of appropriate topical dressings which play an important role in correcting the underlying causes of the wounds and thus, aiding during the healing process. Wound dressings have developed from natural origin agents that basically covered the wound to advanced materials that are particularly planned to provide specific influences on a wound. An open wound is a favorable niche for microbial colonization. The majority of infected wounds are regularly polymicrobial because of being contaminated by the pathogens that are originated from the nearby milieu [1,2]. Nowadays, bacterial contamination of skin wounds is responsible for high rates of morbidity and mortality [3,4]. Recently, the researchers have greatly focused on chitosan (CS) as a substitute for petroleum derivative polymer due to its distinctive properties such as availability from natural sources, biodegradability, biocompatibility and having hemostatic properties [^5-9^]. On the other hand, CS suffers from certain limitations such as inadequate processability and solubility in water, poor mechanical strength, low antibacterial properties, flexibility and low stability in conventional solvents [^10-12^]. In this regard, to overcome these problems, CS is typically used with other materials together rather than in pure form [11] and thus chitosan-based nanocomposite scaffolds have been developed. CS for wound dressing applications can be combined with other nanomaterials to achieve a dressing with improved healing ability, strength and flexibility. Graphene is a material with possible applications in several fields [^13-15^], however due to low solubility in both organic and aqueous solvents, the use of its hydrophilic graphene derivatives such as graphene oxide (GO) for biomedical applications has been suggested. The outstanding properties of GO including large surface area, excellent physical and mechanical properties, high dispersibility, functionality and acceptable biocompatibility highlight its widespread applications [16]. Moreover, recently GO nanostructures have attracted significant interest from researchers in order to manufacture polymeric nanocomposites with improved mechanical properties [^17-21^]. The mechanical properties of GO make it a possible candidate to incorporate into wound dressing providing flexibility and strength.

Untreated wounds present an extended inflammation and constant infections, besides numerous types of drug-resistant bacterial biofilms that are frequently available [13,22,23]. General antibiotics have caused resistance in the wound site and pose a heightened risk to universal public health. It is crucial to develop alternative materials such as inorganic antimicrobial agents to control this resistance. These agents can be normally added to the matrix of wound dressing. Among metal-based active agents and inorganic materials, zinc oxide (ZnO) nanoparticles have gained considerable attention in the clinical view. ZnO nanoparticles due to having heat strength, being safe, presenting extensive antibacterial activity against different bacteria, showing less toxicity and dispersing effects can be easily incorporated into wound dressings giving them unique physicochemical and antibacterial properties [^24-30^]. Traditional wound dressings are naturally dry and, as a result, cannot create a moist environment conducive to wound healing. Additionally, they lack antibacterial properties. Currently, film dressings are garnering significant interest due to their flexibility, ease of application and ability to allow some moisture evaporation while acting as a protective barrier against external contaminants [31]. Notably, chitosan-incorporated film dressings exhibit enhanced mechanical and physical properties compared with pure films. It is of interest for investigators to find hybrid materials with particular physical and chemical compositions for incorporation with CS and consequently to have an advanced wound dressing with improved antibacterial activity and acceptable flexibility [32,33]. The objective of our study is to design a wound dressing for the treatment of infected wounds. Here, an innovative wound dressing is developed by incorporating GO and ZnO into the CS matrix by casting method convening the dressing, higher mechanical properties and improved antibacterial activity while having the ability to keep a moist environment and absorb some wound exudates as well as being biocompatible, non-allergic and non-irritant. Our fabricated polymeric films (hereafter CS–ZnO–GO film) were flexible, uniform and stress resistant. Notably, CS–ZnO–GO film that is locally applied on infected wounds could present antibacterial activity and could promote infected wound healing *in vivo*, thus it may be suitable for direct application to damaged infected wounds.

## Materials & methods

### Materials

Graphite, sulfuric acid (H_2_SO_4_, 96%), potassium permanganate (KMnO_4_), nutrient broth, tryptic soy broth, hydrochloric acid (HCl, 37%), glacial acetic acid, glycerol anhydrous, D-Glucose and polyethylene glycol (PEG-200) were purchased from Merck. Sodium nitrate (NaNO_3_), polysorbate 80, 3-(4,5-Dimethylthiazol2-yl)-2,5-diphenyltetrazolium bromide (MTT), peptone from casein, peptone from soybeans, freund's complete and incomplete adjuvant and hydrogen peroxide (H_2_O_2_, 30%) were from Sigma-Aldrich. Chitosan was from Chitoclear, Iceland, and ZnO (NPs) was obtained from US Research Nanomaterials, Inc., USA (ZnO, 99%, 10–30 nm and nearly spherical). Fetal bovine serum (FBS), bovine serum albumin (BSA), and Roswell Park Memorial Institute medium (RPMI) were purchased from Gibco. L929 cells were obtained from Cells Bank at Avicenna Research Institute. The animals were purchased from Pasteur Institute of Iran.

### Characterization

Fourier transform infrared (FTIR) spectra were obtained using Equinox-55 (Bruker, WI, USA) and were in the range of 3500–400 cm^-1^. FTIR spectroscopy helps identify specific chemical bonds and molecular structures in the material, providing insights into its composition and potential interactions between components. Structural studies and compositional features of the flakes were examined by an x-ray diffractometer (XRD; Rigaku D/max-3C, Japan). The morphology and dimension of the films and materials were investigated using atomic force microscopy (AFM; Dualscope/Rasterscope C26, DME, Denmark). Thermogravimetric analysis of the films was determined by a Thermo Gravimetric Analyzer (TGA-60, SHIMADZU, Japan). Thermal weight loss (TG) and derivate (DTG) were investigated in the temperature range between 50 and 800 °C and a heating rate of 10 °C/min. The mechanical stability of the films was measured with an Instron Tester 6025.

### Synthesis of nano-graphene oxide by Hammers method

Graphene oxide nanosheets were produced by slightly modified Hammers method [34]. Initially, 0.5 g of graphite powder and 0.5 g of sodium nitrate were placed into a 100 ml round bottom flask in an ice bath. The mixture was maintained for 10 min at 0 °C for cooling. Sodium nitrate is used as an oxidizing agent to facilitate the subsequent reactions. Placing the flask in an ice bath is essential to prevent rapid and uncontrolled reactions, as subsequent steps involve strong oxidants. Then, 50 ml of concentrated sulfuric acid (H_2_SO_4_, 96%), was added to the mixture and was homogenized for 30 min. Then, 3.5 g of potassium permanganate gradually was added to the reaction flask over 1 h, and the reaction temperature was controlled below 20 °C. The gradual addition of potassium permanganate controls the reaction rate, preventing excessive heat generation. Maintaining the temperature below 20 °C ensures safety and control during this exothermic reaction. The reaction flask was placed in a water bath of 35 °C and stirred for 2 h. Afterward, the bath temperature was set to 95 °C and 45 ml of deionized water was slowly added into the paste like product. Then, 27.5 ml of deionized water is added quickly, followed by the dropwise addition of 1.5 ml of hydrogen peroxide (H_2_O_2_, 30%), resulting in the formation of a yellowish brown suspension. The produced solid powders were centrifuged at 7000 rpm for 10 min and the precipitation was washed three times with diluted HCl (3%), and it was further washed using deionized water and centrifuging at the same condition. Exfoliation of graphite oxide was approached by sonication in deionized water at room temperature for 1 h resulting in uniform GO dispersions. Then, GO solution was poured into a plate; the lid was closed and then placed in the freezer at -80 °C. When the sample was completely frozen, it was placed in a freeze-drier for 40 h and then the produced graphene oxide powder was stored in a vacuum desiccator.

### Preparation of nanocomposite films

GO (0.1 g) was dispersed in distilled water (100 ml) by using an ultrasonic probe for 20 min. ZnO nanoparticles (0.3 mg) were added to the GO dispersed solution. Then, GO and ZnO solutions were mixed for 15 min in an ultrasonic bath. Then, CS (3 g) was added to this mixture and the mixture was stirred at room temperature (500 rpm). An appropriate amount of acetic acid (1% w/v) was then added to obtain a uniform gel. The mixture was stirred for 30 min in an ultrasonic bath (30 min). After that, PEG 200 (6% w/v) was added dropwise to the mixture and then stirred for 48 h at 500 rpm. Finally, the mixture was treated in an ultrasonic bath for 30 min, and every 25 ml of the mixture was poured into a Petri dish and then dried in an oven at 37 °C for 48 h.

### Absorption & mechanical properties

Absorption tests were conducted to ascertain the quantity of fluid absorbed by the samples within a specific time frame. The absorption properties of the dressing were examined using BS EN 13726-1: 2002 and other related references [35]. A dressing (1 cm × 1 cm) was immersed in the test solution containing 8.298 g of NaCl and 0.367 g of CaCl_2_ dissolved in one liter of deionized water that represented a pseudo-wound exudate. Following incubation at 37 °C and different periods, the dressing was removed and weighed, then the rate of absorbency was calculated. The purpose of studying mechanical properties is to evaluate the difficulty of handling the wound dressing. For example, the poor mechanical strength of film dressing makes them difficult to handle. Furthermore, a wound dressing is essential to be strong and flexible that able to cover the wound surface during the recovery process. Mechanical strength can be determined by measuring the tensile strength and elongation at break of the wound dressing. The incorporation of other materials into the wound dressing can impact the mechanical properties. The mechanical properties of the films, such as tensile strength and elongation at the point of break, were assessed following the ASTM D882-18 standard and relevant references [36]. The determined parameters were reported as maximum load at break (MPa) and percentage of length extension at rupture (%), respectively.

### Antimicrobial properties of film

The antibacterial activity of the films was determined according to ISO 22196: 2011 [37]. The testing solution contained a suspension of 1.5 × 10^8^ CFU/ml inoculum of *Staphylococcus aureus* (ATCC 6538/PTCC 1112) or 1 × 10^8^ CFU/ml of *Pseudomonas aeruginosa* (ATCC 9027/PTCC 1074) or 1.5 × 10^8^ CFU/ml of *Escherichia coli* (ATCC 8739/PTCC 1330). The samples including our produced films and untreated polymeric films (surface area of 50 mm × 50 mm) were prepared in a separate sterile dish and were inoculated with 400 μl of the inoculums, covered with a piece of parafilm (40 mm × 40 mm), the inoculums were gently pressed down onto film so that they spread to the edges. The viable number of bacteria on the different studied surfaces on films and Hartmann hydrofilm dressing (Paul Hartmann AG. 89522 Heidenheim, Germany) (as a control) were determined in duplicate after 0 and 24 h of incubation at 37 °C. Bacteria were recovered from the different films by adding 10 ml of soya casein digest lecithin polysorbate broth (SCDLP) (peptone from casein [17 g/l], peptone from soybean [3 g/l], NaCl [5 g/l], Na_2_HPO_4_ [2.5 g/l], D-glucose [2.5 g/l], lecithin [1 g/l], polysorbate 80 [7 g/l]) neutralizer. The viable number of bacteria was determined following serial dilutions and enumeration on plate count agar (PCA) container and then antibacterial activity was calculated. The antibacterial activity of the films was determined using the following formula: R=(Ut−U0)−(At−U0)=Ut−At

Where R is considered to be the antibacterial activity; U0 and Ut are the averages of the common logarithm of the number of viable bacteria, in cells/cm^2^, recovered from the untreated sample immediately after inoculation and after 24 h, respectively; At is considered the average of the common logarithm of the number of viable bacteria, in cells/cm^2^, recovered from the treated samples following 24 h.

### Cytoxicity *in vitro*

*In vitro* cytotoxicity assay of the films was investigated according to ISO 10993:2009 [38]. Briefly, our produced film and polyvinyl chloride film as a positive control were cut into 3 cm^2^ and 6 cm^2^ pieces, respectively. A piece (3 cm^2^) of high-density polyethylene (HDPE) was served as a negative control. The pieces were rinsed with sterilized PBS buffer thrice and then placed into separate containers consisting 1 ml of cell-culture medium and then incubated for 72 h in the incubator 37 °C to prepare liquid extracts. 1 × 10^4^ of L929 cells were grown in RPMI 1640 supplemented with 10% fetal bovine serum (FBS) and 1% penicillin/streptomycin (pen/strep), in 96-well plates (1 × 10^4^ cells/well). The cells were treated with different concentrations of films extracts and the positive control, accompanied with negative control and culture medium, as a blank control. After 24 h incubation of the plates, the culture medium was carefully removed and 50 μl of the MTT solution (5 mg/ml) was added to each well and the plates were further incubated for 3 h. Finally, the produced formazan crystals were dissolved in 100 μl of DMSO, then the optic density (OD) values of the solubilized formazan crystals are determined using a microplate reader equipped with a 570 nm filter.

### *In vivo* studies (sensitization & irritation)

Sensitization to films was investigated using 15 guinea pigs (ten for test and five for control) of body weight in the range 300–350 g and consistent with ISO 10993-10:2010 [39]. In brief, the physiological saline extract of the test materials and control (physiological saline alone) was injected and after 6 days of induction the tested regions were treated with 10% SDS, on day 21, the challenge test was carried out and after 1 day, all dressings were removed. The appearance of the challenged skin sites for test and control groups were observed at 24 and 48 h following removal, and the skin reactions for erythema were graded and recorded. Skin reactions were graded according to a standard known for sensitizing agents based on ISO recommendations [39], the grades were recorded as follows: 0 (no visible change), 1 (discrete or patchy erythema), 2 (moderate and confluent erythema) and 3 (intense erythema and/or swelling).

The irritation test was performed according to ISO 10993-10:2010 [39]. In brief, three New Zealand white rabbits weighing not less than 2 kg were acclimatized for a minimum of 7 days before testing. The dorsal area of the rabbits was shaved avoiding any abrasion 24 h before treatment, the test extract and control (0.5 ml) were spread on sterile gauze patches (2.5 cm × 2.5 cm) and applied on the back side of each rabbit that was then wrapped with the bandages. For controls, physiological saline was used and applied on the contralateral sides of rabbit. After 4 h, the dressings were removed and the sites were observed for signs of erythema and edema after 24, 48 and 72 h. Signs of erythema and edema formation were both scored from 0 to 4, which were based on the scoring system for skin reaction mentioned in ISO standard recommendations [39].

### Mouse full-thickness skin defect study

The Avicenna Research Institutional Animal Care and Use Committee approved the study protocol (approved no: IR.ACECR.AVICENNA.REC 1397.026). Healthy mice were housed individually and acclimatized for a week before experimenting. Mice were anesthetized with a standard dose of Ketamine/Xylazine and surgical areas were shaved with an electric razor then the operation site of the skin was cleaned with alcohol. Following that, a murine-infected full-thickness skin defect wound model was created. Briefly, on the back of mice, 6-mm diameter full-thickness wounds were excised with a biopsy punch and the wounds were photographed immediately using a digital camera. The mice were randomly divided into the infected control group and CS–ZnO–GO nanocomposite film group. Both groups were inoculated with an appropriate concentration of *S. aureus* suspension. The wound of infected control group was then covered with sterile gauze and another group with our fabricated film dressing. After surgery, animals were allowed free cage activity. The number of bacteria on the wounds on the last day was quantified using the plate count method. For the histopathological study, on the last day the skin wound specimens were harvested, fixed with formaldehyde, embedded in paraffin, cut at a thickness of 5 μm and then stained with H&E.

### Statistical analysis

All the data are presented as the mean value SEM. Statistical significance was determined using Student's *t*-test analysis and statistically significant differences are shown with **p 0.01, ***p < 0.001 and ****p < 0.0001.

## Results

### GO flake characteristics

**Figure 1.**
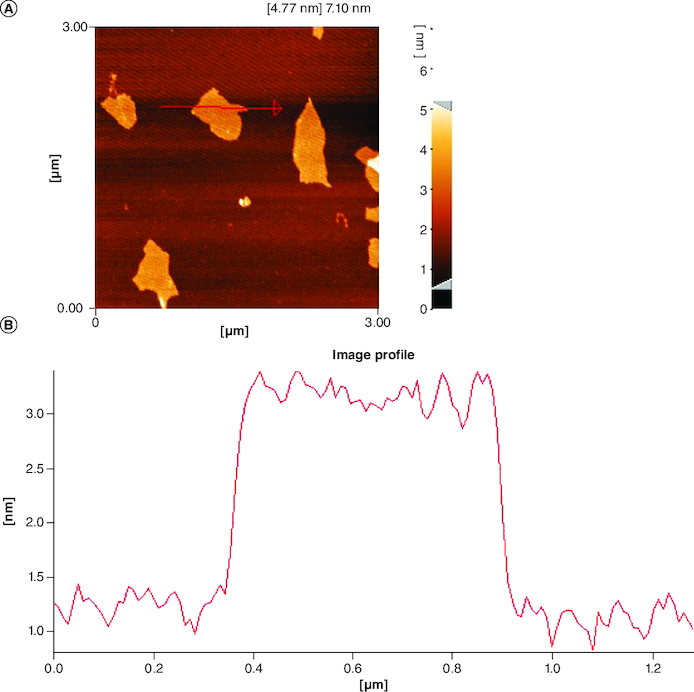
Atomic force microscopy image of graphene oxide flakes and height profiles of graphene oxide nanosheets. **(A)** Atomic force microscopy lateral image of graphene oxide. **(B)** The height profiles of graphene oxide. AFM: Atomic force microscopy; GO: Graphene oxide.

**Figure 2.**
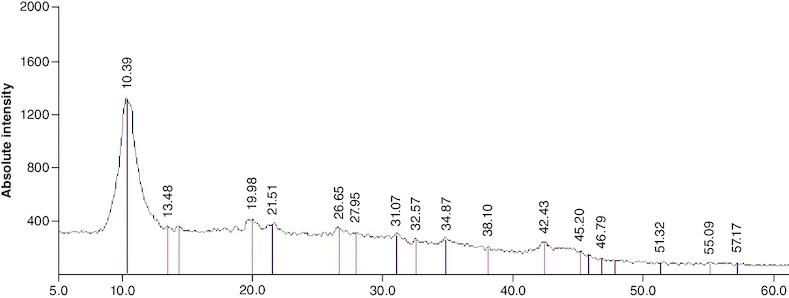
Powder x-ray diffraction patterns of graphene oxide.

Following the synthesis of GO sheet, the size and thickness of single-layer GO flakes were measured using AFM. AFM enables the measurement of surface roughness and the observation of surface features. As shown in Figure 1, the thickness of the synthesized flakes was detected to be ∼2 nm with a lateral dimension of about 300–600 nm, demonstrating that the flakes are monolayered. The x-ray diffraction pattern of graphene oxide synthesized by modified Hummer's method is shown in Figure 2. A sharp peak at 2θ = 10.39° is corresponding to the (001) diffraction peak of disordered GO.

### Characterization of the nanocomposite by Fourier Transform Infrared studies

**Figure 3.**
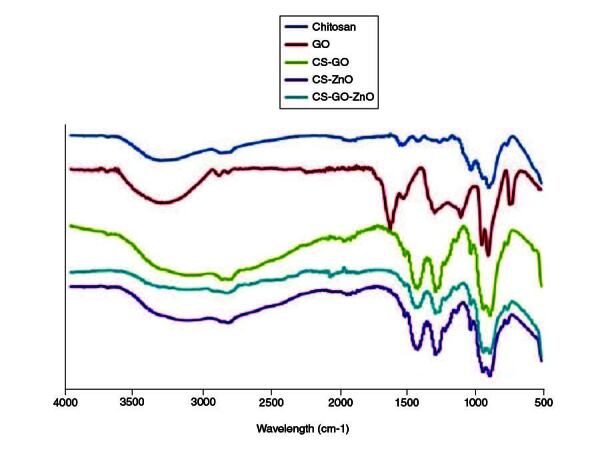
Fourier transform infrared spectra of CS, GO, ZnO, CS–ZnO CS–GO and CS–ZnO–GO hybrid structure. CS: Chitosan; CS–ZnO CS–GO: Chitosan–Zinc oxide Chitosan–Graphene oxide; CS–ZnO–GO: Chitosan–Zinc oxide–Graphene oxide; GO: Graphene oxide; ZnO: Zinc oxide.

FTIR spectral analyses were carried out to identify the successful synthesis of the nanocomposite and the interaction between GO, ZnO and CS as shown in Figure 3. In our results, the FTIR spectra of chitosan, GO nanoparticles, CS–GO, CS–ZnO and our fabricated nanocomposite were compared with provide a vision into the nanoparticle-nanocomposite interfaces. In the spectrum of graphene oxide, the characteristic carboxylic group C=O stretch peak appeared at 1733 cm^-1^, and the deformation peak of the O–H bond of water appeared at 1624 cm^-1^ [40]. The peaks at 1049 and 1414 cm^-1^ correspond to C–O–C stretching vibrations and C–OH stretching of the sp^2^ carbon skeletal network, respectively, while peaks located at 1733 and 3405 cm^-1^ correspond to C–O stretching vibrations of the -COOH groups and O–H stretching vibration, respectively. These functional groups make GO highly hydrophilic and render it dispersible. In the FTIR spectrum of the CS sample, two characteristic absorbance bands were observed that include the C=O stretching vibration of –NHCO- and the N–H bending vibration of NH_2_, appearing at 1659 and 1581 cm^-1^, respectively [41]. The FTIR spectrum of the CS–GO composite showed a combination of characteristics similar to that of the CS and GO. Absorption bands corresponding to NH_2_ at 1581 cm^-1^ in CS and peaks corresponding to C=O stretch of carboxylic group at 1733 cm^-1^ in GO are disappeared in the spectrum of CS–GO, which is due to the interaction between CS and GO. Moreover, the C=O characteristic stretching band of the amide at 1659 cm^-1^ was shifted to 1619 cm^-1^ due to the hydrogen bonding between CS and the oxygenated functional groups in GO and also the electrostatic interaction between polycationic CS and the negative charge on the surface of GO. These interactions may have a strong impact on the mechanical properties, swelling and degradation of the composite film. In the FTIR spectrum of the CS–ZnO composite, compared with chitosan IR spectrum, O–H stretching vibration peak in CS–ZnO is broader 3600–2500 cm^-1^, which indicated the interaction between these groups and ZnO [42]. The absorption peaks at 2925, 2870 cm^*-*1^ are attributed to asymmetric stretching of CH_3_ and CH_2_ of chitosan. While the absorption peaks at 1619 and 1063 cm^*-*1^ are ascribed to bending vibration of –NH_2_ group and C–O stretching group. The FTIR spectra of the CS–ZnO–GO composite features characteristic peaks of both chitosan, GO and ZnO due to interaction between their functional groups. The formation of GO-chitosan composite may be due to a reaction between the epoxy groups on the surface of GO and the amino groups (NH_2_) on the surface of chitosan. The absence of the peak at 1733 cm^-1^ in the FTIR spectra of the CS–ZnO–GO composite is likely due to the low mass ratio of GO to chitosan. This peak corresponds to C=O in carboxylic acid and carbonyl moieties in GO, but it is too weak to be observed under these conditions [43,44]. However, the special characteristic of the IR spectra of CH–GO–ZO composite is flattening of O–H stretching band at 3000 cm^-1^, showing the decreased stretching of free OH and/or NH related to the binding interactions between GO and ZnO and CS [45].

### Thermal analysis of the film

**Figure 4.**
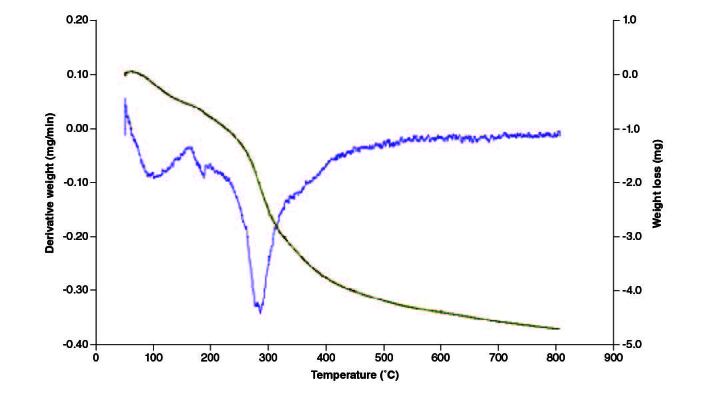
TGA-DTG curve representing thermal decomposition of CS–ZnO–GO nanocomposite film.

TGA was carried out to examine the thermal stability and decomposition behavior of the nanocomposite film. TGA measures weight alterations as a function of temperature and offers information on thermal degradation, decomposition temperatures and the existence of volatile components. This analysis assists in assessing the film's stability under different temperature conditions. Thermal decomposition of films occurred in three main stages in an argon atmosphere. As shown in the thermograms (Figure 4), loss of water molecules presents in films occurred until 175 °C (10%) [46]. The second weight-loss step as a sharp and considerable peak in DTG was between 230 and 300 °C (30%) due to thermal degradation of polymer chains in chitosan, elimination of glycosidic units of polymer chains, and elimination of volatile components of nanocomposite [47]. After 400 °C, there was a continuous weight loss of 70% up to 800 °C, this could be mainly due to the degradation and decomposition of the polymer backbone involving decomposition of CH and the reduction of GO [48].

### Swelling & mechanical properties of the films

We evaluated the absorbency rate to determine the relevance of the films to the fluid. The absorbency values of pure chitosan and our fabricated film were 18.86 and 3.01 g/g^-1^, respectively. This result indicated that our fabricated film could absorb at least three-times the fluid of its dry weight. Following the absorption of fluid, the films could maintain their structural integrity. The fluid absorption capacity of pure chitosan is more than our fabricated film that can be due to crosslinking between chitosan chains through GO nanoparticles. Typically, our fabricated films can be applied to none-to-low-exudating wounds and may correspondingly be proper for moderately exudating wounds.

The mechanical properties of films play an important role in defining the most suitable choice for a certain type of wound. The films should be able to resist the proper stress through their application on specified wounds and represent enough flexibility during exudates absorption preventing their distraction. The stress-strain profiles obtained by tensile testing could show the mechanical performance of CS–ZnO–GO nanocomposites. The results clearly showed that the tensile strength (TS) and elongation at the break (E) of the films were 20.1 ± 0.7 MPa and 36 ± 10%.

### Bactericidal efficiency of the CS–ZnO–GO films

Numerous bacterial agents have been identified as culprits of infections, with *S. aureus*, *E. coli* and *P. aeruginosa* being recognized as the predominant agents responsible for wound infections. We analyzed the antibacterial property of fabricated films against mentioned bacteria. Our results clearly showed significant antibacterial activity of fabricated films. The reported colony numbers for *E. coli* and *S. aureus* were 1 × 10^4^ CFU/ml and 1 × 10^3^ CFU/ml, respectively, while for *P. aeruginosa* no growth was observed.

### Cell viability assay with the extract of CS–ZnO–GO film

The prepared films must be inert and biocompatible to be suitable for wound healing applications. Extracts of the film were applied to determine *in vitro* cytotoxicity in L929 cells. Our film extract revealed no cytotoxic effects after 24 h, as they displayed cell viability rates greater than 90% (93.4 ± 0.4%) that was determined by MTT assay. Cell viability between 70 and 100% illustrates that there were no cytotoxic components in the extract based on ISO 10993-5 standard recommendation [38]. Neither the test extract nor the negative control induced any morphological changes in L929 cells. On the other hand, the viability of L929 cells was significantly decreased in positive controls (1.8 ± 0.3%) compared with negative controls. Taken together, the cell viability of our fabricated films is comparable to that in the negative control and thus our prepared films have no cytotoxic effect.

### Sensitization test results

The guinea pig is the animal of choice for evaluation of allergic contact sensitization, due to its high sensitivity and reproducibility. The allergenicity (skin sensitization) of prepared films was assessed according to ISO standard [39]. The challenged skin sites of test and control animals were observed at 24 and 48 h after removing the dressings. Skin reactions for erythema and edema were scored using numerical grading as mentioned elsewhere [39]. Our results indicated that the physiological saline extract of the control and test animals did not cause any adverse skin reaction such as erythematous or swelling and grade 0 was given to them. Thus, this dressing presented no risk of allergic reaction and can be considered non-allergenic to the skin, according to the recommendations of ISO [28].

### Irritation test results

An important requirement for antimicrobial dressings is being non-irritant and non-reactive with biological tissue at the application site. The skin region of tested rabbit treated with CS–ZnO–GO films demonstrated the normal dermal appearance with no signs of skin erythema or edema and irritating effects or lesion during *in vivo* dermal irritation test. All obtained values for erythema and edema were reported to be zero. There was no difference between the scores of the tested and control groups. According to the recommendations of ISO standard [39], the primary irritation index below 0.4 for irritation test is regarded as negligible. Our fabricated films did not provoke an allergic reaction and had a negligible irritation for animals and thus can be classified as a non-irritant to the skin.

### Wound contraction & histopathological examinations

**Figure 5.**
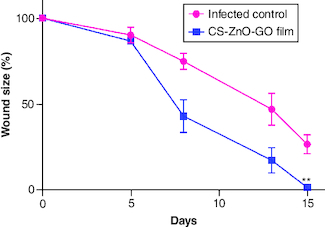
Wound contraction evaluation. Changes in wound size for different groups. **p < 0.01.

**Figure 6.**
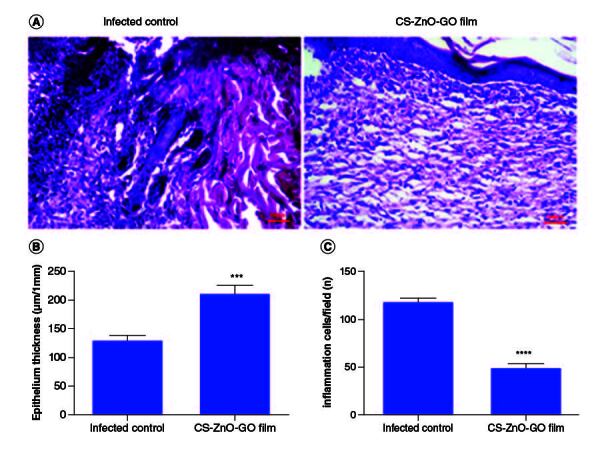
Histopathological examinations. **(A)** Representative H&E stained tissue slices from different groups on day 15. Wound healing statistical data of different groups after 15 days of treatment. (**B)** Epithelium thickness (μm/1 mm). **(C)** Number of inflammation cells/field. ****p < 0.0001; ***p < 0.001. CS–ZnO–GO:Chitosan–zinc oxide–graphene oxide.

The application of our fabricated wound dressing reduced the total *S. aureus* populations in the skin wounds of mice compared with the infected control group. The average CFU/ml estimate for the infected control group was almost 1.6-times higher than wound dressing-treated group on the last day of experiment. The wound size reduction rate was quantitatively determined by measuring the wound area (Figure 5). Initially, no significant difference was detected in the wound size of both groups, however on day 15, the wound size reduction rate in CS–ZnO–GO nanocomposite film-treated group was apparently higher as compared with the infected control group (Figure 5). On the other hand, microscopic assessment using H&E of wounds showed that CS–ZnO–GO nanocomposite film-treated group has different epithelial thickness compared with the infected control group (Figure 6A), as mentioned in the graph, statistically significant difference was observed between the treated and control group, in which control group has thinner epithelium compared with nanocomposite film-treated group (Figure 6B). In addition, there was a reduction in the inflammatory infiltrate in nanocomposite film-treated group compared with the infected control group. The treated group showed a significant decrease in the number of inflammatory cells when compared with control group at 15th days of the wound healing process (Figure 6A & C).

## Discussion

The incorporation of chitosan in wound dressings offers a solution to the limitations of traditional dry dressings. In particular, chitosan-based film dressings excel in terms of their flexibility, ease of application and their capacity to facilitate controlled moisture evaporation while establishing a protective shield against external contaminants. Chitosan's unique properties synergize effectively with other hybrid materials that possess specific physical and chemical compositions, thereby enhancing the mechanical strength of the film. These films have a wide range of applications, including promoting rapid wound healing and preventing infections. Their non-toxic and biocompatible nature makes them promising for wound management. Ongoing research aims to further enhance the performance of these films, with a focus on chitosan-based options as versatile wound dressings. The comprehensive characterization of the CS–ZnO–GO nanocomposite films reveals a multitude of promising features that position these materials for various applications, particularly in wound care and tissue repair.

The synthesis of high-quality single-layer GO flakes with precisely controlled dimensions is a significant achievement. These monolayered GO flakes, with their consistent thickness and lateral dimensions, offer versatility for diverse applications, including advanced wound dressings (Figures 1 & 2). GO sheet surface consists of several oxygen-containing groups, that are much simpler to react with other sets to complete GO functionalization or hybridization with supplementary ingredients.

Our FTIR spectral analysis clearly provided valuable information regarding the interactions within the nanocomposite. FTIR spectra of the CS–ZnO and CS–ZnO–GO composites apparently demonstrated, the binding interactions between chitosan, zinc oxide (ZnO) and GO (Figure 3). Notably, the strong interactions observed between chitosan and GO nanoparticles, as well as with ZnO, underscore the potential for enhanced mechanical properties, swelling behavior and degradation characteristics. These interactions are key to tailoring the composite's performance for specific medical applications.

Thermal analysis highlights the film's controlled decomposition behavior, a critical aspect for applications involving controlled release. The multistage decomposition process, including water loss, polymer chain degradation and polymer backbone decomposition, ensures stability in various environmental conditions (Figure 4). Several factors can affect the fluid absorption of the dressings [49,50]. A dressing absorption capacity stands as a concession among conflicting needs. Our fabricated dressings were designed to resolve the exudate absorbency of occlusive dressing, deprived of conceding on the moist environment that is required for tissue repair [51,52].

The enhanced TS and E values of the film compared with pure chitosan film (data not shown) can be explained by the presence of GO nanoparticles which are as cross-linkers between chitosan chains. Generally, the quality of dispersion and interfacial stress transfer play crucial roles for the production of reinforcing nanocomposites [27,53]. These mechanical properties make the composite film resilient and capable of withstanding the stress associated with application to various types of wounds. Antibacterial wound dressing films should be able to prevent infection caused by bacteria [54,55]. The infection has been reported to postpone the healing progression, to damage wound bed, to rise trauma care and accordingly management charges [56,57]. A variety of bacterial agents have been demonstrated to cause infections and among them *S. aureus*, *E. coli* and *P. aeruginosa* are considered to be the most dominant wound infection causing bacterial agents. The substantial antibacterial activity exhibited by the composite films against common wound infection-causing bacteria, underscores their potential for preventing infections and facilitating wound healing. The synergistic effects between chitosan, ZnO and GO contribute to this robust antibacterial property. The ability to prevent bacterial growth in the wound site can be reflected as an added benefit for our fabricated films.

The biocompatibility of the composite film, as confirmed through cytotoxicity tests, is a fundamental characteristic for its application in medical settings. The absence of cytotoxic effects and the maintenance of cell viability align with ISO standards, ensuring the safety of the film for use in contact with living tissues. Moreover, the results from sensitization and irritation tests on animal models further validate the safety profile of the composite films. The absence of allergic reactions and skin irritation positions these films as non-allergenic and non-irritant to the skin, supporting their potential for use in clinical scenarios.

The application of this fabricated wound dressing significantly reduced the total population of *S. aureus* in mouse skin wounds. This reduction in bacterial load indicates the antimicrobial efficacy of the nanocomposite film. Furthermore, the wound size reduction rate was notably higher in the CS–ZnO–GO nanocomposite film-treated group. This suggests that the wound dressing not only controls infection but also accelerates the wound healing process. Microscopic examination revealed distinct differences in epithelial thickness between the two groups. The nanocomposite film-treated group exhibited a thicker epithelium compared with the infected control group, and this difference was statistically significant. Additionally, the nanocomposite film-treated group displayed a reduction in the inflammatory infiltrate in the wound site. This reduction in inflammation is indicative of the wound dressing's ability to mitigate the inflammatory response during the wound healing process (Figures 5 & 6). Overall, the findings suggest that the CS–ZnO–GO nanocomposite wound dressing effectively reduces wound healing time and promotes a favorable wound healing environment. Its antimicrobial properties, coupled with its ability to enhance epithelial thickness and reduce inflammation, make it a promising candidate for the management of infected wounds. Further research and clinical trials are warranted to validate its safety and effectiveness in practical wound care scenarios.

## Conclusion

Our chitosan-based dressing due to its biodegradability, non-toxicity and antimicrobial properties, can be considered as a possible alternative for traditional dressing in wound healing applications. Conclusively, the modification of GO nanosheets with chitosan not merely developed the dispersion quality of nanosheets and mechanical properties of nanocomposites but also increased the availability of CS to obtain a film with suitable biocompatibility. In addition, due to the synergistic effects of ZnO, the antibacterial activity of the film was significantly increased following the addition of ZnO to GO/CS films. The combination of ZnO and GO nanoparticles with chitosan not only improved antimicrobial activity and healing process but also enhanced the mechanical characteristics of wound materials without aberrant cytotoxicity, while being non-irritant and non-allergenic. Taken together, this nanocomposite film displayed a great potential as a novel multifunctional wound dressing application.
